# Progress in the pathological arena of gynecological cancers

**DOI:** 10.1002/ijgo.13871

**Published:** 2021-10-20

**Authors:** W. Glenn McCluggage

**Affiliations:** ^1^ Department of Pathology Belfast Health and Social Care Trust Belfast UK

**Keywords:** cervix, endometrium, female genital tract, FIGO Cancer Report, gynecological cancer, pathology, tubo‐ovarian, vagina, vulva

## Abstract

This review covers the significant new developments in the pathological classification of gynecological tumors. Many of these were included in the updated World Health Organization Classification of Female Genital Tract Tumours, published in 2020. Topics include the compelling evidence that a large majority of extrauterine high‐grade serous carcinomas arise from the fallopian tube; the Cancer Genome Atlas (TCGA) Classification of endometrial carcinomas; the discovery that most so‐called synchronous endometrial and ovarian endometrioid carcinomas represent metastasis from the endometrium to the ovary; and the division of cervical, vaginal, and vulval carcinomas into clinically meaningful HPV‐associated and HPV‐independent types. Newly described tumor types are covered, including endometrial and ovarian mesonephric‐like adenocarcinoma, uterine sarcoma types associated with specific molecular abnormalities, and gastric (gastrointestinal)‐type adenocarcinomas of the endometrium and vagina. Important molecular events in ovarian sex cord–stromal tumors are also discussed.

## Synopsis

Review of significant new developments in the classification of gynecological tumors; many are included in the 2020 World Health Organization Classification of Female Genital Tract Tumours.

## 1 INTRODUCTION

The updated 2020 World Health Organization (WHO 2020) Classification of Female Genital Tract Tumours (5th edition) was published online and in the traditional “Blue Book” in Autumn 2020.^1^ The present review covers the significant developments and major changes in the classification of gynecological cancers, some of which emanate from the new WHO Classification. In a review such as this, most of the topics cannot be covered in detail and the reader is referred to the key references provided herein.

## 2 WELL‐ESTABLISHED TUBAL ORIGIN OF MOST EXTRAUTERINE HIGH‐GRADE SEROUS CARCINOMAS

It is now well established that a significant majority of extrauterine (primary ovarian, tubal, or peritoneal) high‐grade serous carcinomas (HGSC) arise from the distal fimbrial end of the fallopian tube from a precursor lesion known as STIC (serous tubal intraepithelial carcinoma). The evidence is compelling, both in sporadic cases and cases associated with germline *BRCA* mutations.^2^, ^3^, ^4^, ^5^ Unfortunately, this has not translated uniformly into clinical and pathological practice; in other words, the same extrauterine HGSC in a resection specimen could be categorized as of tubal, ovarian, or peritoneal origin by different pathologists. Although typically not important for management, this has obvious implications for epidemiological reasons and cancer registration and the tubal origin raises the possibility of prophylactic salpingectomy with ovarian preservation in premenopausal women at increased risk of the development of HGSC. Criteria for site assignment in extrauterine HGSC have been proposed^3^, ^4^, ^5^ (Table 1) and the use of these criteria results in a high proportion (approximately 80%) being classified as tubal in origin, while primary peritoneal HGSC are exceedingly rare; this diagnosis should only be made when there is no ovarian parenchymal involvement and no mucosal STIC or HGSC within either tube, both of which should be grossly visible in their entirety and examined in total histologically using a SEE‐FIM (sectioning and extensively examining the fimbriated end of the fallopian tube) protocol.^2^, ^3^, ^4^, ^5^, ^6^ These recommendations have been endorsed by the International Collaboration on Cancer Reporting (ICCR) and in the cancer datasets of the Royal College of Pathologists in the UK and the College of American Pathologists.^6^

**TABLE 1:** Criteria for primary site assignment in extrauterine high‐grade serous carcinomas

Criteria	Primary site	Comment
STIC present	Fallopian tube	Regardless of presence and size of ovarian and peritoneal disease
Invasive mucosal carcinoma in tube, with or without STIC	Fallopian tube	Regardless of presence and size of ovarian and peritoneal disease
One or both fallopian tubes partially or entirely incorporated into tubo‐ovarian mass	Fallopian tube	Regardless of presence and size of ovarian and peritoneal disease
No STIC or invasive mucosal carcinoma in either tube in presence of ovarian mass or microscopic ovarian involvement	Ovary	Both tubes should be clearly visible and fully examined by a standardized SEE‐FIM protocol Regardless of presence and size of peritoneal disease
Both tubes and both ovaries grossly and microscopically normal (when examined entirely) or involved by benign process in presence of peritoneal HGSC	Primary peritoneal HGSC	This diagnosis should only be made in specimens removed at primary surgery prior to any chemotherapy; see below for samples following chemotherapy
HGSC diagnosed on small sample, peritoneal/omental biopsy or cytology, OR HGSC examined after chemotherapy	Tubo‐ovarian	Note: this should be supported by clinicopathological findings to exclude mimics, principally uterine serous carcinoma

Abbreviations: HGSC, high‐grade serous carcinoma; SEE‐FIM, sectioning and extensively examining the fimbriated end; STIC, serous tubal intraepithelial carcinoma.

## 3 NO EVIDENCE FOR “FIELD EFFECT” IN HIGH‐GRADE SEROUS CARCINOMA

It was previously thought that HGSC could arise at multiple independent sites through some form of “field effect.” However, it has been convincingly shown that HGSC at different sites are clonal, with one site representing the primary and the others being metastatic. Almost all extrauterine HGSC exhibit *TP53* mutation as an early founder event, and mutational analysis of ovarian and peritoneal HGSC with concurrent STIC has shown these to harbor identical *TP53* mutations in the vast majority of cases.^7^, ^8^ The demonstration of an identical *TP53* mutation in tumors at different sites is strong evidence for clonality, as the probability of an identical mutation occurring simultaneously at multiple sites is extremely low. It can be summarized that there is irrefutable evidence that almost all HGSC arises from a single tumor clone and that multiple foci of disease do not result from a multifocal origin or a field effect.^7^, ^8^ Similarly, it has been shown that cases of uterine serous carcinoma with involvement of the fallopian tube (even when confined to the tubal mucosa) mostly represent tubal metastases and not independent primary tubal lesions.^9^

## 4 THE CANCER GENOME ATLAS (TCGA) MOLECULAR CLASSIFICATION OF ENDOMETRIAL CARCINOMAS

In 1983, in a seminal and widely cited paper, Bokhman proposed that there were two broad types of endometrial carcinoma, type I and type II.^10^ Broadly speaking, type I carcinomas (prototypically endometrioid‐type) arise in perimenopausal or early postmenopausal women, are low‐grade, typically early‐stage neoplasms arising on a background of atypical hyperplasia and are positive with hormone receptors. Type II carcinomas (prototypically serous‐type) arise in elderly postmenopausal women, are high‐grade, typically advanced‐stage neoplasms arising in atrophic endometria and are negative with hormone receptors. However, although useful as a broad concept, it was always clear that there is too much overlap in the clinical and pathological features in many individual tumors and the Bokhman classification never gained widespread acceptance among pathologists.

The current 2020 WHO Classification of endometrial carcinomas,^1^ like prior classifications, is based on morphology and in practice pathologists often use a variety of immunohistochemical markers to assist in classifying problematic neoplasms. However, especially with “high‐grade” endometrial carcinomas (serous, clear cell, grade 3 endometrioid, mixed, undifferentiated, and dedifferentiated carcinoma and carcinosarcoma), there is significant interobserver variation even among expert gynecological pathologists.^11^ For example, in one study, three observers examined 56 high‐grade endometrial carcinomas and in 20 of 56 (35.8%) cases there was a major disagreement, including no consensus regarding the major subtype diagnosis or even whether a component of high‐grade carcinoma was present.^11^

In 2013, TCGA published a seminal comprehensive molecular study of 373 endometrial carcinomas; the study was restricted to endometrioid, serous, and mixed endometrioid**–**serous carcinomas with no inclusion of other high‐grade carcinomas.^12^ Tumors were analyzed using a variety of modalities, including exome sequencing, somatic copy number alteration, whole genome sequencing, mRNA expression, protein expression, microRNA expression, and DNA methylation. The study revealed that endometrial carcinoma is a complex disease consisting of four intrinsic molecular types: *POLE* (ultramutated), microsatellite instability (MSI, hypermutated), copy‐number low (also referred to as microsatellite stable or no specific molecular profile), and copy‐number high (serous‐like). It was demonstrated that the four molecular types are of prognostic significance, with *POLE* tumors having the best prognosis (even though they often look high grade morphologically) and copy‐number high the worst.^12^ Regarding the percentages of the four molecular types, copy‐number low is the most prevalent accounting for approximately 39%, followed by MSI hypermutated (28%), copy‐number high (26%), and *POLE* ultramutated (7%).

Since the delineation of the four molecular types and the demonstration of prognostic significance, there has been an explosion of studies investigating how to incorporate molecular typing into routine reporting of endometrial carcinomas, and a simplified combined morphological–molecular classification (such as the ProMisE classifier^13^) is likely to be incorporated into routine practice in the near future. The recommendation in WHO 2020 is to integrate microscopic features with molecular characteristics in regions where the resources are available.^1^ Molecular classification is likely to be of particular value in high‐grade endometrioid carcinomas by picking out the *POLE*‐mutated (good actors) and the copy‐number high (poor actor) neoplasms.^14^ *POLE* mutation analysis may not be necessary in typical low‐grade endometrioid carcinomas since *POLE* mutations are not common in this group and these neoplasms would not qualify for adjuvant therapy unless they are advanced stage at diagnosis. Typical serous carcinomas may also not require *POLE* mutation analysis. *CTNNB1* mutation analysis (or perhaps beta‐catenin immunohistochemistry as a surrogate), L1 cell adhesion molecule (L1CAM), and p53 may be of value in low‐grade endometrioid carcinomas in identifying those cases likely to have a worse outcome.^15^ An obvious drawback is that currently *POLE* mutation analysis is not available in most pathology laboratories and, as such, development of an appropriate infrastructure will be required; this will likely entail centralization of testing in a limited number of reference laboratories. It is also clear that the molecular classification will be complementary to morphology since parameters such as depth of myometrial invasion, lymphovascular space invasion and cervical and nodal involvement, which are prognostically significant, can only be identified on morphological examination.

Recent studies have shown that TCGA classification also has prognostic significance in other endometrial carcinoma types such as carcinosarcoma, clear cell carcinoma, undifferentiated/dedifferentiated carcinoma and neuroendocrine carcinoma.^16^, ^17^, ^18^

## 5 SYNCHRONOUS ENDOMETRIAL AND OVARIAN ENDOMETRIOID CARCINOMAS

It is not uncommon for a patient to have an endometrioid carcinoma in the endometrium and one, or occasionally both, ovaries and a variety of clinicopathological parameters are used by pathologists to distinguish between synchronous independent primaries and metastasis usually from the endometrium to the ovary. Especially when both neoplasms are low grade, it has long been assumed that most of these represent dual primaries and the prognosis has been assumed to be good, although there are few studies with long‐term follow‐up. It has recently been demonstrated that such neoplasms involving the uterine corpus and ovary are clonal and probably represent metastasis from one site to the other, usually from the endometrium to the ovary.^19^, ^20^, ^21^ Thus, there is a dilemma in that, although molecularly these are clonal neoplasms, and most represent Stage IIIA endometrial carcinomas, the prognosis is thought to be good and there is potential for overtreatment with the unnecessary administration of adjuvant therapy. In WHO 2020 it is recommended that conservative management is undertaken and management should be as for synchronous neoplasms when the following four criteria are met: (1) both tumors are low grade; (2) <50% myometrial invasion; (3) no involvement of any other site; (4) absence of extensive lymphovascular invasion at any location.^1^

## 6 CHANGES TO ENDOMETRIAL AND OVARIAN CARCINOMA CLASSIFICATION IN WHO 2020

In WHO 2020 there have been few changes to the classification of ovarian and endometrial carcinomas.^1^ The category of mixed carcinoma of the ovary has been reintroduced having been deleted from the prior classification, although it is stressed that mixed carcinomas of the ovary are extremely uncommon. The category of seromucinous ovarian carcinoma has been removed since most of these are considered to represent morphological variants of endometrioid carcinoma.^22^, ^23^ The category of mesonephric‐like adenocarcinoma (MLA) (discussed below) has been added to the classification of both ovarian and endometrial carcinomas. A new category of gastric (gastrointestinal)‐type mucinous carcinoma of the endometrium has been introduced; these are aggressive neoplasms with identical morphology and immunophenotype to gastric‐type adenocarcinomas of the cervix.^24^

### 6.1 Mesonephric‐like adenocarcinoma (MLA)

Mesonephric carcinomas are rare neoplasms that most commonly arise in the uterine cervix. They are assumed to arise from normal or hyperplastic mesonephric remnants. Recently a “new” entity has been described, termed MLA; these neoplasms may arise both in the endometrium and the ovary^25^, ^26^, ^27^ and they have been included in the 2020 WHO Classification.^1^ These neoplasms exhibit considerable morphological, immunophenotypic, and molecular similarity to true mesonephric carcinomas. The term MLA is used since although these neoplasms closely resemble mesonephric carcinomas, other parameters suggest a Müllerian origin. In the uterine corpus these neoplasms appear to arise from the endometrium and spread into the myometrium; none of the neoplasms involved the myometrium without or with minimal endometrial involvement, as might be expected with a true mesonephric carcinoma arising from mesonephric remnants. In the ovary these neoplasms are often associated with endometriosis. Moreover, in both the uterine corpus and ovary, these neoplasms have not been associated with mesonephric remnants. In the original publication^25^ it was debated whether these represent true mesonephric carcinomas or Müllerian carcinomas that closely mimic mesonephric carcinoma and it was proposed that the term MLA be used until the histogenesis is firmly established.^25^

It is clear that MLAs are aggressive neoplasms with a propensity for distant spread, especially to the lungs. In a multi‐institutional study, the clinicopathologic features of 44 MLAs and 25 MLAs of the uterine corpus and ovary, respectively, were studied.^27^ The majority of MLAs presented at advanced stage and developed recurrences, most commonly distant. It was recommended in this study that MLA should not be graded but be regarded automatically as high grade.

## 7 NEW DEVELOPMENTS IN UTERINE SARCOMAS

Up until relatively recently, almost all uterine sarcomas were considered to represent leiomyosarcoma, low‐grade endometrial stromal sarcoma, undifferentiated sarcoma, and rare “heterologous” sarcomas, such as rhabdomyosarcoma. However, the last few years have witnessed the description of several “new” uterine sarcoma types, such as high‐grade endometrial stromal sarcomas associated with *YWHAE*‐*NUTM2A*/*B* or *BCOR* genetic abnormalities, undifferentiated sarcomas associated with *SMARCA4* mutation and sarcomas associated with neurotrophic tropomyosin receptor kinase (*NTRK)* rearrangements.^28^, ^29^, ^30^, ^31^, ^32^ Predominantly these neoplasms were discovered using sophisticated molecular techniques, such as next generation sequencing, which have revealed novel diagnostic molecular events. Various other molecular abnormalities have also been reported in uterine sarcomas and with the increasing availability of these molecular techniques it is inevitable that additional “new” entities will be reported in the near future. This will result in diminution of the category of undifferentiated sarcoma.

## 8 CHANGES IN CLASSIFICATION OF CERVICAL SQUAMOUS LESIONS

The 2014 WHO Classification of cervical squamous cell carcinomas (SCC) divided these neoplasms into essentially meaningless morphological types, such as keratinizing, nonkeratinizing, basaloid, warty, papillary, squamotransitional, verrucous, and lymphoepithelioma‐like. These represent morphological variations rather than tumor types and, in practice, most pathologists did not use these categories, which suffered from lack of reproducibility and were of no prognostic significance. The 2020 WHO Classification categorizes cervical SCC into HPV‐associated and HPV‐independent types.^1^ Table 2 compares the 2014 and 2020 WHO Classifications of cervical SCC. This is a welcome extension of the well‐established trend to categorize SCC at many sites into HPV‐associated and HPV‐independent types, for example in the vulva and vagina (see below) and in extragenital organs such as the head and neck region. In most sites, the division into HPV‐associated and HPV‐independent SCC is prognostically significant with a generally better prognosis for HPV‐associated neoplasms. The prognostic significance in the cervix is not yet established given that HPV‐independent SCC are uncommon, comprising approximately 7% of cervical SCC.^33^ Given the marked preponderance of HPV‐associated neoplasms, it is controversial as to whether confirmatory studies to confirm an HPV association (most commonly p16 immunohistochemistry but also HPV testing) should be undertaken in all cases, especially since there are currently no management or prognostic implications. It is established that morphology is not always reliable in distinguishing between HPV‐associated and HPV‐independent SCC, for example in the vulva, and this is also the case in the cervix.^33^ p16 staining (block‐type in HPV‐associated and negative or mosaic‐type in HPV‐independent neoplasms) is much more reliable at predicting HPV status. As such, an argument can be made for undertaking p16 staining in all cervical SCC and if staining is not block type, an HPV‐independent neoplasm should be considered and HPV testing undertaken using highly sensitive molecular techniques. An alternative is to undertake ancillary testing only in those cases where the morphology (well‐differentiated keratinizing) raises the possibility of an HPV‐independent neoplasm or when corroborative evidence of an HPV‐associated neoplasm, such as adjacent high‐grade squamous intraepithelial lesion is absent. The 2020 WHO Classification also includes a category of SCC, not otherwise specified (NOS) to be used in settings where p16 staining or HPV testing is not available.^1^

**TABLE 2:** Comparison of the 2014 and 2020 WHO classifications of cervical squamous cell carcinoma

WHO 2014	WHO 2020^1^
Squamous cell carcinoma, usual type Keratinizing Nonkeratinizing Papillary Basaloid Warty Verrucous Squamotransitional Lymphoepithelioma‐like	Squamous cell carcinoma, HPV‐associated Squamous cell carcinoma, HPV‐independent Squamous cell carcinoma, NOS

Abbreviation: NOS, not otherwise specified.

## 9 CHANGES IN CLASSIFICATION OF CERVICAL GLANDULAR LESIONS

Traditionally (including in WHO 2014), cervical adenocarcinomas were classified based on morphology and were divided into clinically meaningless and poorly reproducible categories with no biological basis, including villoglandular, endometrioid, and serous. Analogous to cervical SCC, cervical adenocarcinomas are now categorized in WHO 2020 into HPV‐associated and HPV‐independent types (Table 3). Most cervical adenocarcinomas are HPV‐associated but a higher percentage than SCC (about 15%–20%) are HPV‐independent.^34^, ^35^, ^36^, ^37^, ^38^, ^39^ HPV‐independent cervical adenocarcinomas typically present at higher stage and have a worse prognosis.^34^, ^35^, ^36^, ^37^, ^38^, ^39^ The 2020 WHO Classification also divides adenocarcinoma precursor lesions into HPV‐associated and HPV‐independent types, the latter including gastric‐type adenocarcinoma in situ and atypical lobular endocervical glandular hyperplasia.^1^, ^40^

**TABLE 3:** Comparison of the 2014 and 2020 WHO classifications of cervical adenocarcinoma

WHO 2014	WHO 2020^1^
Endocervical adenocarcinoma, usual type Mucinous carcinoma NOS Mucinous carcinoma, gastric type Mucinous carcinoma, intestinal type Mucinous carcinoma, signet ring cell type Villoglandular carcinoma Mesonephric carcinoma Serous carcinoma Clear cell carcinoma Serous carcinoma Adenocarcinoma NOS	HPV‐associated cervical adenocarcinoma (includes several subtypes; see text) HPV‐independent cervical adenocarcinoma Gastric type Mesonephric type Clear cell type Other adenocarcinomas

Abbreviations: NOS, not otherwise specified.

HPV‐associated adenocarcinomas are typified by easily identifiable mitotic figures and apoptotic bodies and almost always exhibit diffuse block‐type immunoreactivity with p16. Subtypes of HPV‐associated adenocarcinoma include usual type and mucinous type; the former encompasses villoglandular and micropapillary variants and mucinous type encompasses stratified mucin producing carcinoma, intestinal, signet ring, and NOS variants.^1^, ^41^

HPV‐independent types of cervical adenocarcinoma are gastric type (the most common), mesonephric, and clear cell.^36^, ^37^, ^38^, ^39^ As discussed, these typically have a worse prognosis than HPV‐associated adenocarcinomas and are almost always p16 negative or focally positive (nonblock‐type immunoreactivity).

Serous carcinoma of the cervix is not included in the 2020 WHO Classification. Most tumors previously diagnosed as such represent usual‐type HPV‐associated cervical adenocarcinomas with papillary architecture and high‐grade nuclear features or direct involvement by or a drop metastasis of a tubo‐ovarian or endometrial serous carcinoma.

## 10 VAGINA AND VULVA

Similar to the cervix, vaginal and vulval SCC are now divided into HPV‐associated and HPV‐independent types in WHO 2020.^1^ This replaces those types included in the prior classification, namely keratinizing, nonkeratinizing, papillary, basaloid, warty, and verrucous; the reasons underlying these changes are exactly analogous to those discussed in the section on cervical SCC. Although primary vaginal SCC are considerably more uncommon than in the cervix and the vulva, there is convincing evidence that, similar to other sites, HPV‐independent neoplasms have a worse prognosis.^42^, ^43^ It is recommended that the type of vaginal SCC (HPV‐associated or HPV‐independent) be documented on the pathology report. However, as at other sites, a morphological diagnosis of SCC NOS is acceptable when resources required to differentiate between the two, such as p16 immunohistochemistry and HPV testing, are not available.

Similarly in the vulva, traditional histologic typing of vulval SCC has been superseded by HPV status as the major determinant of classification. HPV‐independent SCC have a worse prognosis with significantly worse recurrence‐free and overall survival compared with HPV‐associated SCC.^44^, ^45^, ^46^, ^47^, ^48^ There is also growing evidence that HPV‐independent SCC are less responsive to radiotherapy.^47^ The majority of HPV‐associated SCC exhibit basaloid or warty morphology, while HPV‐independent SCC tend to be keratinizing; however, a significant percentage of cases (15%–20%) show overlapping morphologic features. While the nature of any adjacent precursor lesion may be useful in helping to determine the HPV status, in practice, ancillary testing (p16 or HPV testing) is necessary given the overlap in morphology. As in the cervix and vagina when HPV status cannot be confidently determined or resources are not available to undertake ancillary testing, a diagnosis of SCC NOS is acceptable, although this is not recommended. Most, but not all, HPV‐independent vulval SCC are associated with *TP53* mutations.^49^ However, a proportion are *TP53* wild type and there is growing evidence that these may have an intermediate prognosis between HPV‐associated SCC (best prognosis) and HPV‐independent *TP53* mutated neoplasms (worst prognosis).^49^

There have also been changes to the classification of primary vaginal adenocarcinomas with the description of new entities such as HPV‐associated and gastric‐type adenocarcinomas,^50^, ^51^ both of which may arise in adenosis; the latter are aggressive primary vaginal adenocarcinomas morphologically and immunophenotypically identical to their cervical counterparts. Broadly, with some minor differences, the classification of primary vaginal adenocarcinomas mirrors that in the cervix.^50^, ^51^

## 11 NEW DEVELOPMENTS IN OVARIAN SEX CORD–STROMAL TUMORS AND MISCELLANEOUS NEOPLASMS

In WHO 2020, the classification of ovarian sex cord–stromal tumors is largely unchanged from the prior classification.^1^ The category of gynandroblastoma (mixed sex cord–stromal tumor) has been reintroduced having been removed from the prior classification. This is one area of ovarian pathology where significant advances have been made in recent years. Sex cord–stromal tumors represent a heterogenous group of uncommon neoplasms that, when they exhibit classical morphology, are relatively easy to diagnose. However, there may be considerable morphological overlap between the different tumor types, and immunohistochemistry, while useful in confirming a sex cord–stromal tumor, is of minimal value in distinguishing between the different tumor types. Recent significant advances (see Table 4) include the demonstration that adult granulosa cell tumors contain somatic *FOXL2* mutations in well over 90% of cases,^52^ while a significant proportion of moderately and poorly differentiated Sertoli–Leydig cell tumors contain *DICER1* mutations; these may be somatic or germline, the latter signifying DICER1 syndrome.^53^, ^54^ Ongoing studies are elucidating the molecular events in several other tumor types within the sex cord–stromal category. For example, microcystic stromal tumor contains *CTNNB1* or less frequently *APC* mutations and is occasionally an extracolonic manifestation of familial adenomatous polyposis.^55^, ^56^ In problematic cases, demonstration of the appropriate molecular abnormality assists in tumor classification.

**TABLE 4:** Recently described molecular events in ovarian sex cord–stromal and miscellaneous neoplasms

Tumor type	Molecular event
Adult granulosa cell tumor	Somatic *FOXL2* mutations
Sertoli–Leydig cell tumor	Somatic or germline *DICER1* mutations
Microcytic stromal tumor	*CTNNB1* or *APC* mutations
Small cell carcinoma of the ovary of hypercalcemic type	Somatic or germline *SMARCA4* mutations

Small cell carcinoma of the ovary of hypercalcemic type (SCCOHT), which is included in the category of miscellaneous ovarian neoplasms in WHO 2020, has been shown to be characterized by deleterious germline or somatic mutations in a single gene *SMARCA4* ^57^, ^58^, ^59^ in well over 90% of cases. *SMARCA4* is part of the SWI/SNF complex, which is implicated in the pathogenesis of a growing number of other malignancies. Demonstration of this mutation and/or loss of immunohistochemical staining with SMARCA4 (BRG1) may, in the correct morphological context, be crucial in the diagnosis of this highly aggressive neoplasm.^60^, ^61^ It is recommended that all patients diagnosed with SCCOHT should be referred for germline *SMARCA4* mutation testing.^62^, ^63^

## CONFLICTS OF INTEREST

The author has no conflicts of interest to declare.

## AUTHOR CONTRIBUTIONS

The author is responsible for the design and writing of the paper.
